# Global Gene Expression and Docking Profiling of COVID-19 Infection

**DOI:** 10.3389/fgene.2022.870836

**Published:** 2022-04-11

**Authors:** Almas Jabeen, Nadeem Ahmad, Khalid Raza

**Affiliations:** ^1^ Department of Bioscience, Jamia Millia Islamia, New Delhi, India; ^2^ Department of Computer Science, Jamia Millia Islamia, New Delhi, India

**Keywords:** coronavirus, gene expression profiling, pathological biomarkers, infection and immune system, differentially expressed genes network

## Abstract

Coronavirus is an enclosed positive-sense RNA virus with club-like spikes protruding from its surface that causes acute respiratory infections in humans. Because it is considered a member of the complex pathogen group, it has been found to infect different host species and cause a variety of diseases. So far, it has been discovered that it may affect the immune, infection, and inflammatory systems, leading to the hypothesis that the immune and inflammatory systems (signaling pathways and components) fail to control infection, opening the door to look for potential targets primarily in these systems. The study’s main purpose is to identify highly overexpressed genes and their functional implications as a result of COVID-19 infection, as well as to investigate probable infections, inflammation, and immune systems to better understand the impact of coronavirus infection. We explored the genes and pathways mostly linked with infection, inflammation, and the immune systems using the datasets available for COVID-19 infection gene expression compendium. NFKBIA, FN1, FAP, KANK4, COMP, FAM101B, COL1A2, ANKRD1, TAGLN, SPARC, ADAM19, OLFM4, CXCL10/11, OASL, FOS, APOBEC3A, IFI44L, IFI27, IFIT1, RSAD2, NDUFS1, SRSF6, HECTD1, CBX3, and DDX17 are among the genes that may be impacted by infection, according to our findings. The functional changes are mainly associated with these pathways TNF, cytokine, NF—kB, TLR, TCR, BCR, Foxo, and TGF signaling pathways are among them and there are additional pathways such as hippo signaling, apoptosis, estrogen signaling, regulating pluropotency of stem cells, ErbB, Wnt, p53, cAMP, MAPK, PI3K—AKT, oxidative phosphorylation, protein processing in endoplasmic reticulum, prolactin signaling, adipocytokine, neurotrophine signaling, and longevity regulating pathways. Moreover, we have also explored the potential herbal drug (apigenin, quercetin, and resveratrol) targets for the top-rated genes based on the overall analysis where we observe that quercetin and resveratrol as most effective.

## Introduction

The Centers for disease Control and Prevention (CDC) is monitoring a global health threat caused by coronavirus, and an outbreak of respiratory illness caused by this unique coronavirus was first discovered in Wuhan, Hubei Province, China, in recent years (Dean 1994; Marra et al., 2003; Hassan et al., 2020; Wu et al., 2020; Zhu et al., 2020). The current epicenter of the outbreak is in Wuhan, China, but other cases have been discovered in a growing number of other areas throughout the world, and investigations are continuing (Med and FAAS 2019; Hamid et al., 2020; Lu et al., 2020; Zhao et al., 2020).

Coronavirus is a positive-sense RNA virus with club-like spikes protruding from its surface that is encapsulated (Li 2013; Nishiga et al., 2020; Sharin et al., 2020). Although it is most commonly associated with acute respiratory infections in humans, it is a challenging pathogen due to its ability to infect multiple host species and cause a variety of diseases (Bernardes et al., 2020; Singhal 2020; Stolfi et al., 2020; Zhao et al., 2021). Because wild animals frequently come into contact with humans, zoonotic diseases are becoming more widespread. SARS—CoV and MERS—CoV are zoonotic diseases that cause severe respiratory disorders in humans (Báez-Santos et al., 2015; Venkataraman and Frieman 2017; Jackson et al., 2020; Singhal 2020; Zhao et al., 2020).

Coronaviruses can be found in a number of animals, including camels and bats, and they can evolve and infect people in rare circumstances, spreading the virus from one person to the next. MERS—CoV and SARS—CoV-2 are two more recent human coronaviruses that have been linked to serious illness. The other five coronaviruses are 1) 229E (alpha), 2) NL63 (alpha), 3) OC43 (beta), 4) HKU1 (beta), and 5) SARS—CoV (Sironi et al., 2015; Al-Hazmi 2016; Rabaan et al., 2017; Chen et al., 2019; Med and FAAS 2019). A variety of circumstances, according to early study, could contribute to an outbreak like the one seen in Wuhan, China. 1) The first and most essential component is the Wuhan seafood market, which has been identified based on early data. As a result, we can deduce that the virus spreads from animals to humans. Services for diagnosis and treatment should be offered across the country, especially in high-risk areas. Massive public awareness campaigns about the 2019-nCoV infection should be carried out on a large scale in order to inform the general public about the preventative measures that should be taken. 2) Interactions between animals and humans are common (Cao et al., 2020; Djalante et al., 2020; Laxminarayan et al., 2020; Sharin et al., 2020). Although it appears that little is known about the entire process of coronavirus harm at this time, several studies involving coronaviruses have been published so far, spanning from genome mapping to gene expression profiling to mutational profiling, as well as epigenetic investigations. The most current study, which focuses mostly on COVID—19, was also released, and it looked into the full genome mapping and phylogenetic investigation and finding the possible solutions in terms of diagnosis (Cao et al., 2020; Hartung and Aktas 2020; Hirano and Murakami 2020; MD et al., 2020; Moujaess et al., 2020; Wiersinga et al., 2020; Wu et al., 2020).

Coronavirus replication occurs in the cytoplasm of host cells, generating inflammatory responses, and the exact processes are currently unknown. COVID-19 symptoms range from mild to severe disease, and the explanation of this variation in infection response is unknown (Hu et al., 2020; Hartung and Aktas 2020; Hirano and Murakami 2020; de las Heras et al., 2020; Schultze and Aschenbrenner 2021; Tay et al., 2020). Understanding the immunological mechanisms at work at various stages of the colonization process could be essential to understanding these disparities. We used a dataset downloaded from a public database to conduct a multi-tissue (nasal, buccal, and blood) gene expression investigation of immune-related genes in patients with various COVID-19 severity levels and healthy controls. Furthermore, the GEO (Gene Expression Omnibus) dataset of infected and uninfected human cell lines was obtained in order to investigate particular information on the gene expression pattern for infection, inflammation, and the immune system as a result of coronavirus infection and replication. We compared control to infected and uninfected to infected samples from different datasets that were either infected or not infected from this dataset. There are two types of samples: one with infection and the other either uninfected or control, so we compared control to infected and uninfected to infected. We employed two sets of DEGs (differentially expressed genes) and enriched pathways for these analyses, and then compared the DEGs and enriched pathways.

For the self-management of COVID-19 illness, self-isolation, relaxation, water, and the use of non-steroidal anti-inflammatory drugs (NSAIDs) only in situations of severe fever are now recommended (COVID-19) (Silveira et al., 2020). Many patients are asked to add symptomatic or adjuvant therapy, such as herbal medicines, to their regimen. In the current COVID-19 pandemic, to assess the benefits and risks of some herbal drugs commonly used to treat respiratory illnesses as an adjuvant treatment. There is no evidence-based particular therapy for COVID-19 yet, and when enough multi-site clinical data becomes available, the true efficacy and safety of current therapeutic options will need to be examined further (Hirano and Murakami 2020; Hu et al., 2020; Silveira et al., 2020; Tay et al., 2020). Thus, we performed herbal drug docking profiling for the top-ranked DEGs in order to suggest viable medicines against the COVID-19 infection. Furthermore, we chose three potential herbal medications, apigenin (Caltagirone et al., 2000), quercetin (Caltagirone et al., 2000), and resveratrol (Imran et al., 2020), and did a docking research using fold change, *p*-value, and network-level analysis against the top-ranked genes.

## Methods

The study’s major purpose was to see how coronavirus infection altered human gene expression and function, hence gene expression datasets from healthy and infected samples were acquired from the Gene Expression Omnibus (GEO). The data comes from GSE17400 (Yoshikawa et al., 2010), which contains 28 samples, and the platform was GPL570 [HG-U133 Plus 2], Affymetrix Human Genome U133 Plus 2.0 Array. We only selected samples that were infected and infected only for the comparative investigation since we were primarily interested in understanding the potential difference between gene expression patterns in infected and uninfected situations. There were also three different time point samples, which we noted. We’ve also added other datasets for cross-verification.

GSE183071 (https://www.ncbi.nlm.nih.gov/geo/query/acc.cgi?acc=GSE183071) sample details (Total number of samples = 156): At the same timepoints, blood, nasal epithelium, and saliva samples were taken from patients and controls. Following the manufacturer’s instructions, RNA was extracted using the PAXgene blood miRNA extraction kit (Qiagen). Oragene CP-190 kit was used to collect saliva and nasal epithelial samples (DNA Genotek). Patients and healthy controls self-identified as being of South-European ancestry, and cases were categorised by severity of illness at the time of sample collection: severe (ICU admission), moderate (non-ICU but admitted to hospital), and mild (non-ICU but admitted to hospital) (domiciliary lockdown patients with mild symptoms or asymptomatic). 1) whole blood from 41 patients and 13 controls, 2) nasal epithelium from 38 patients and 11 controls, and 3) saliva from 41 patients and 12 controls.

GSE17400 (Yoshikawa et al., 2010) sample details (Total number of samples = 27): To characterize the dynamic, geographic, and temporal variations in gene expression caused by SARS-CoV, confluent 2B4 cells grown in T-75 flasks were infected with SARS-CoV (MOI = 0.1) or left uninfected (control) for 12, 24, and 48 h. Because 2B4 cells were permissive to the productive infection of Dhori virus (DHOV), a member of the Orthomyxoviridae family within the Thogotovirus genus, resulting in robust responses of IFNs and other pro-inflammatory mediators, they also established parallel cultures of DHOV-infected 2B4 cells (MOI = 0.1) for the comparative analysis of global gene expression elicited by SARS-CoV- versus DHOV-in. They have performed the investigation in triplicate for mock-, SARS-CoV-, and DHOV-infected cultures at each time point to satisfy the minimum number required for statistical techniques, resulting in a total of 27 arrays. In a nutshell, supernatants from differentially treated cultures were taken at 12-, 24-, and 48 h for analysis of viral yields and cytokine profile, while the cells were subjected to total RNA extraction using an RNAqueous-4PCR kit and following the manufacturer’s protocol. Purified RNA samples were sent to our core facility, where they were converted to cDNA, biotin-labeled, and hybridized to 27 Affymetrix Human Genome U133 Plus 2.0 “Gene Chips,” each of which had 54,675 probe set identifiers representing over 47,400 transcripts identifying 38,500 well-characterized genes, as well as various internal controls (Affymetrix, Santa Clara, CA). Mock-infected cells were compared to SARS-CoV or DHOV-infected cells at each time point.

By splitting samples into two groups: control and infected, and uninfected and infected, and then calculating fold changes and *p*-values, we were able to compile a list of DEGs using GEO2R (*p*-value cutoff 0.05). Off course, we have calculated the false discovery rate (FDR) but here, *p*-value have been used as cutoff. For pathway analysis, we used the KEGG database and developed our own algorithm for pathway and network analysis (Kanehisa et al., 2007; Kanehisa et al., 2009; Kanehisa et al., 2011).

After researching DEGs and enriched pathways, the next step was to understand the network and the connections between the genes inside the DEG network. The network was shown using cytoscape (Shannon et al., 2003) and the FunCoup database. MATLAB coding was also used for figure plotting and analysis. The primary idea of the FunCoup network database is that it predicts four different forms of functional coupling or links, such as protein complexes, protein-protein physical interactions, metabolic, and signaling pathways (Alexeyenko and Sonnhammer 2009; Bajrai et al., 2021b; Bajrai et al., 2021a; Eldakhakhny et al., 2021; Kamal et al., 2020; Mobashir et al., 2012; Mobashir et al., 2014; Warsi et al., 2020). Swiss-dock (http://www.swissdock.ch) (Grosdidier et al., 2011; Kumar et al., 2020) was utilized for docking analysis, with PubChem (https://pubchem.ncbi.nlm.nih.gov/), UniProt (https://www.uniprot.org/), and swiss-model (https://swissmodel.expasy.org) as supporting databases (Kumar et al., 2020). From docking analysis, we have collected Gibb’s free energy. Gibbs free energy is a thermodynamic potential which are used to calculate the maximum reversible work. The network-level and needed analytic scripts were written in MATLAB, as were the results, which included the number of connections per gene and the genes belonging to different numbers of pathways (Kamal et al., 2020; Helmi et al., 2021).

## Results

**FIGURE 1:**
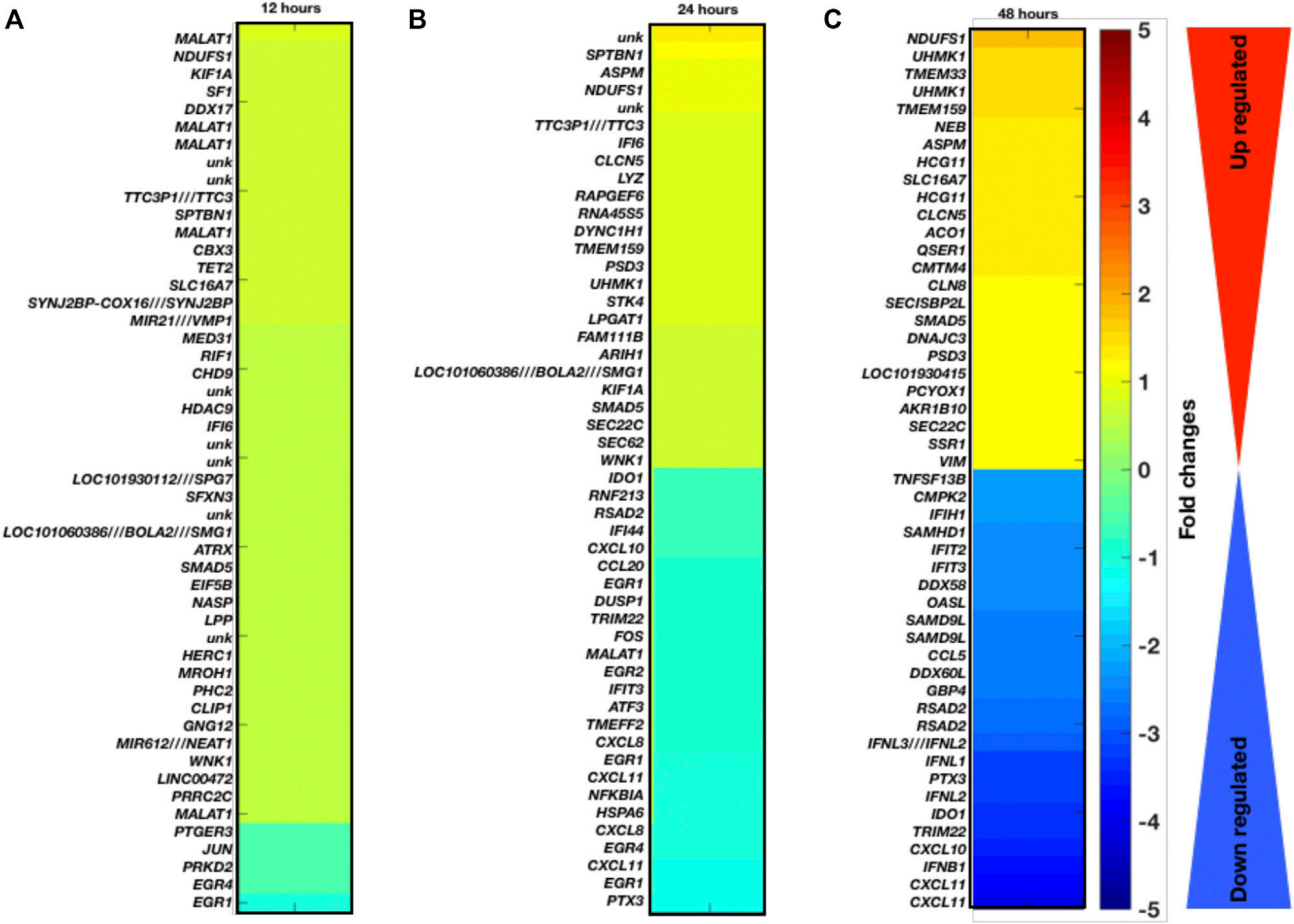
Gene expression profiling and enriched pathways. Top-ranked 50 DGEs based on fold changes and *p*-values at different infection time period **(A)** 12 h, **(B)** 24 h, and **(C)** 48 h.

**FIGURE 2:**
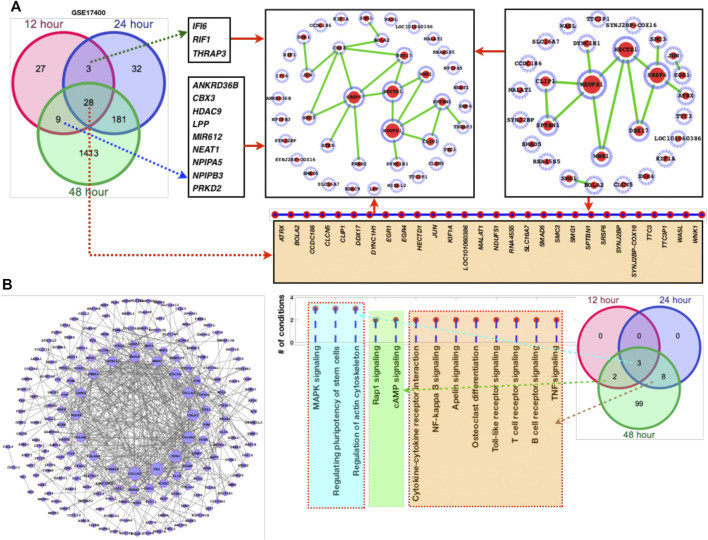
DEGs network and their respective fold changes and p-values. **(A)** Venn diagram followed by the DEGs network, **(B)** DEGs (181 genes shared between 24 and 48 h) with their fold changes, **(C)** the enriched KEGG pathways followed by the venn diagram to display the alterations at different time points.

Temporal gene expression profiling and differentially expressed genes as a result of coronavirus infection: Here, we have explored the gene expression pattern for the gene expression datasets of 12 h, 24 h, and 48 h post coronavirus infection. Basically both these datasets are from GSE17400 which are either infected or uninfected (mock infected). In this case, the samples have been grouped as control (mock) and infected at three different time points. For all these three data, we have calculated the DEGs and enriched pathways and performed comparative analysis (Figures 1A–C). For all these three time points, we have plotted the heatmaps with the respective fold changes for the top 50 DEGs. MALAT1, NDUFS1, KIF1A, SF1, DDX17, EGR1, EGR4, PRKD2, JUN, PTGER3, PRRC2C, SPTBN1, ASPM, NDUFS1, TTC3P1 (TTC3), IFI6, CLCN5, LYZ, RAPGEF6, RNA45S5, DYNC1H1, TMEM159, CXCL8, EGR1, CXCL11, NFKBIA, HSPA6, CXCL8, EGR4, CXCL11, EGR1, PTX3, NDUFS1, UHMK1, TMEM33, UHMK1, TMEM159, NEB, ASPM, HCG11, SLC16A7, HCG11, CLCN5, RSAD2, IFNL3 (IFNL2) IFNL1, PTX3, IFNL2, IDO1, TRIM22, CXCL10, IFNB1, and CXCL11 are among the top-ranked genes in three time points 12 h, 24 h, and 48 h. These genes display maximum fold changes at different time points of infection. The potential point we observe in these three time points of infection is that increased duration of COVID-19 infection leads to further aggravation in gene expression which is obvious from the fold changes of the DEGs. Here, it appears that the fold changes of the DEGs are increased further with increased duration of infection. Based on venn diagram (Figure 2A), we observe that there are 28 common DEGs for all the three time points, 3 DEGs common between 12 and 24 h, 181 DEGs common between 24 and 48 h, and 9 DEGs common to 12 and 48 h. This leads to the conclusion that higher time COVID-19 infection could affect the genes expression at much higher rates than the initial COVID-19 infection.

Furthermore, we have also added larger dataset GSE183071 which contains 156 samples for better clarification and crosschecking of the predicted DEGs. Here, also most of the DEGs of GSE17400 overlaps the DEGs of GSE183071 which means that irrespective of the datasets majority of these genes are abnormally affected as a result of COVID-19 infection. After analyzing the DEGs and comparing with the other dataset of COVID-19, network analysis has been performed for the sets of genes (Figure 2). Among these networks, we observe that NDUFS1, SRSF6, HECTD1, CBX3, and DDX17 are highly connected (Figure 2A). Since, the overall number of DEGs were quite high for the common DEGs between 24 and 48 h, so we have separately plotted the network of it. More details have been provided in supplementary dataset which contains the genes fold changes, *p*-values, enriched pathways, and docking scores (S1).

Dominantly altered genes dominantly controls infection, inflammation, and immune system associated functions and pathways: As discussed in previous section and shown in Figure 1 that the top-ranked DEGs appear to be mostly inferring to infection and inflammatory pathways. So we have performed the enriched pathways analysis for the list of DEGs at different time points and for different datasets. In Figure 2C, it is clearly shown that there are three pathways which are dominantly altered in all the three time points, eight altered pathways common between 24 and 48 h, and only two pathways common between 12 and 48 h. There is no 12 h or 24 h specific pathway while there are huge number of pathways specific to 48 h. Thus, we could conclude that the initial infection stage has less functional disturbance while higher time of infection could further deteriorate the functional systems and pathways. In this venn diagram, we see that there are eight pathways which are common between 24 and 48 h are purely the part of infection and inflammatory systems. Most of these pathways directly infer the immune system and the source major infection. TNF, cytokine, NF—kB, TLR, TCR, and BCR signaling pathways are among them. Moreover, we have also compared this dataset pathways list with the second dataset enriched pathways and observe that these pathways are common there too. There are additional pathways such as hippo signaling, apoptosis, estrogen signaling, regulating pluripotency of stem cells, ErbB, Wnt, p53, cAMP, MAPK, PI3K—AKT, oxidative phosphorylation, protein processing in endoplasmic reticulum, prolactin signaling, adipocytokine, neurotrophine signaling, and longevity regulating pathways. The additional pathways are the three junctions (tight, gap, and adherens), CAMs (cell adhesion molecules), cell cycle, HIF-1, metabolic processes, VEGF, and focal adhesion (Table 1).

**TABLE 1:** Enriched pathways for GSE183071 COVID-19 dataset DEGs.

Enriched Pathways	*p*-values
KEGG_04010_MAPK_signaling_pathway	5.42E-20
KEGG_04060_Cytokine-cytokine_receptor_interaction	5.42E-20
KEGG_04064_NF-kappa_B_signaling_pathway_-_Homo_sapiens_(human)	5.42E-20
KEGG_04145_Phagosome	5.42E-20
KEGG_04151_PI3K-Akt_signaling_pathway_-_Homo_sapiens_(human)	5.42E-20
KEGG_04210_Apoptosis	5.42E-20
KEGG_04380_Osteoclast_differentiation	5.42E-20
KEGG_04514_Cell_adhesion_molecules_(CAMs)	5.42E-20
KEGG_04612_Antigen_processing_and_presentation	5.42E-20
KEGG_04620_Toll-like_receptor_signaling_pathway	5.42E-20
KEGG_04630_Jak-STAT_signaling_pathway	5.42E-20
KEGG_04640_Hematopoietic_cell_lineage	5.42E-20
KEGG_04650_Natural_killer_cell_mediated_cytotoxicity	5.42E-20
KEGG_04660_T_cell_receptor_signaling_pathway	5.42E-20
KEGG_04662_B_cell_receptor_signaling_pathway	5.42E-20
KEGG_04668_TNF_signaling_pathway_-_Homo_sapiens_(human)	5.42E-20
KEGG_04068_FoxO_signaling_pathway_-_Homo_sapiens_(human)	4.11E-19
KEGG_04722_Neurotrophin_signaling_pathway	4.11E-19
KEGG_04917_Prolactin_signaling_pathway	1.56E-16
KEGG_04920_Adipocytokine_signaling_pathway	1.56E-16
KEGG_04015_Rap1_signaling_pathway_-_Homo_sapiens_(human)	4.78E-14
KEGG_04810_Regulation_of_actin_cytoskeleton	4.78E-14
KEGG_04014_Ras_signaling_pathway_-_Homo_sapiens_(human)	7.65E-13
KEGG_04066_HIF-1_signaling_pathway_-_Homo_sapiens_(human)	7.65E-13
KEGG_04670_Leukocyte_transendothelial_migration	7.65E-13
KEGG_04071_Sphingolipid_signaling_pathway_-_Homo_sapiens_(human)	1.15E-11
KEGG_04510_Focal_adhesion	1.15E-11
KEGG_04621_NOD-like_receptor_signaling_pathway	1.15E-11
KEGG_04550_Signaling_pathways_regulating_pluripotency_of_stem_cells	1.61E-10
KEGG_04610_Complement_and_coagulation_cascades	1.61E-10
KEGG_04622_RIG-I-like_receptor_signaling_pathway	1.61E-10
KEGG_04370_VEGF_signaling_pathway	2.09E-09
KEGG_04611_Platelet_activation	2.09E-09
KEGG_04672_Intestinal_immune_network_for_IgA_production	1.58E-08
KEGG_04664_Fc_epsilon_RI_signaling_pathway	2.51E-08
KEGG_04115_p53_signaling_pathway	2.76E-07
KEGG_04360_Axon_guidance	2.76E-07
KEGG_04919_Thyroid_hormone_signaling_pathway	2.76E-07
KEGG_04921_Oxytocin_signaling_pathway	2.76E-07
KEGG_04012_ErbB_signaling_pathway	2.76E-06
KEGG_04072_Phospholipase_D_signaling_pathway_-_Homo_sapiens_(human)	2.48E-05
KEGG_04310_Wnt_signaling_pathway	2.48E-05
KEGG_04350_TGF-beta_signaling_pathway	2.48E-05
KEGG_04392_Hippo_Signaling_Pathway	2.48E-05
KEGG_04520_Adherens_junction	2.48E-05
KEGG_04750_Inflammatory_mediator_regulation_of_TRP_channels	2.48E-05
KEGG_04623_Cytosolic_DNA-sensing_pathway	1.08E-04
KEGG_04110_Cell_cycle	1.98E-04
KEGG_04512_ECM-receptor_interaction	1.98E-04
KEGG_04912_GnRH_signaling_pathway	1.98E-04
KEGG_03050_Proteasome	1.39E-03
KEGG_04024_cAMP_signaling_pathway_-_Homo_sapiens_(human)	1.39E-03
KEGG_04211_Longevity_regulating_pathway	1.39E-03
KEGG_04540_Gap_junction	1.39E-03
KEGG_04666_Fc_gamma_R-mediated_phagocytosis	1.39E-03
KEGG_04910_Insulin_signaling_pathway	1.39E-03
KEGG_04915_Estrogen_signaling_pathway	1.39E-03
KEGG_00230_Purine_metabolism	8.33E-03
KEGG_04022_cGMP-PKG_signaling_pathway_-_Homo_sapiens_(human)	8.33E-03
KEGG_04120_Ubiquitin_mediated_proteolysis	8.33E-03
KEGG_04140_Regulation_of_autophagy	8.33E-03
KEGG_04141_Protein_processing_in_endoplasmic_reticulum	8.33E-03
KEGG_04371_Apelin_signaling_pathway_-_Homo_sapiens_(human)	8.33E-03
KEGG_04916_Melanogenesis	8.33E-03
KEGG_04020_Calcium_signaling_pathway	4.17E-02
KEGG_04080_Neuroactive_ligand-receptor_interaction	4.17E-02
KEGG_04144_Endocytosis	4.17E-02
KEGG_04261_Adrenergic_signaling_in_cardiomyocytes	4.17E-02
KEGG_04530_Tight_junction	4.17E-02
KEGG_04723_Retrograde_endocannabinoid_signaling	4.17E-02
KEGG_04914_Progesterone-mediated_oocyte_maturation	4.17E-02

Network-level understanding of commonly DEGs: After analyzing the DEGs and the enriched pathways, we have created a network of the common DEGs between the two datasets 1 and 2 and individually mapped their respective fold changes, *p*-values, and the inferred pathways (Figures 2A–C). In GSE47962 DEGs network, we observe that COL1A1/2, FN1, TPM1, COL5A1, CALD1, and COL3A1 appear control large number of genes and thus more pathways. In addition, we have also presented a list of pathways where these genes belong or are the component of it. Similar to common enriched pathways, there were more than 60 pathways (Table 1) enriched for the second COVID-19 dataset GSE183071. The common DEGs are the component of these pathways and for ranking the genes based on the pathways shared by these genes, we have presented a list of genes based on the number of shared pathways by these genes (Figure 2C). MAPK pathway, actin cytoskeleton regulation, and pluripotency of stem cells regulation share all the three time points which means they directly associate additional pathways and these pathways exclusively known for infection and immune systems leading to the conclusion that corona virus infection directly damage immune system and those cellular systems which are known to balance and counter external infection.

**FIGURE 3:**
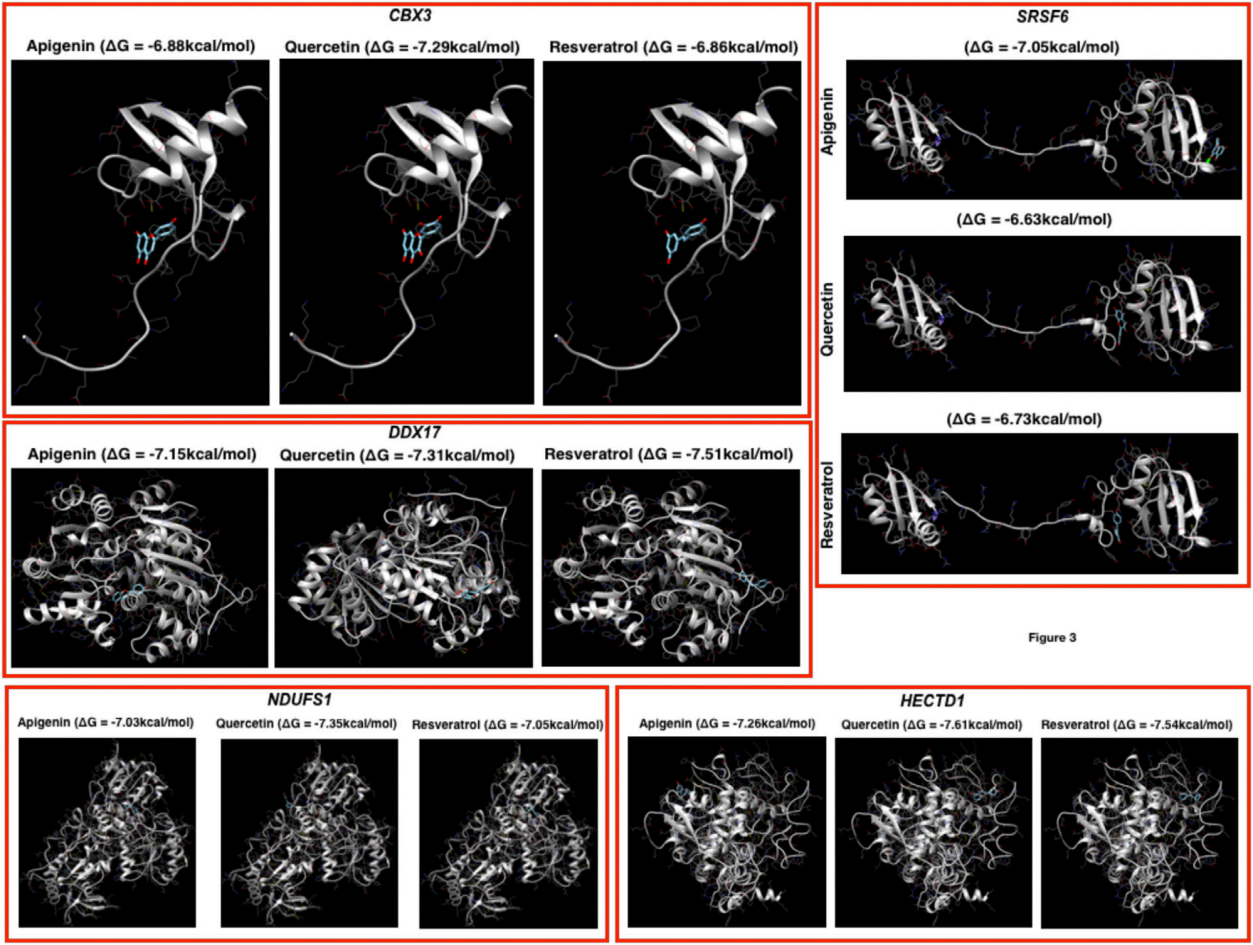
Docking profiling. Here, the docking has been performed by using the three herbal drugs (apigenin, quercetin, and resveratrol) against the top five genes.

For these top-ranked genes based on the number of pathways where they belong, subnetworks have been presented and from the subnetworks, it clearly appears that NDUFS1, HECTD1, SRSF6, DDX17, and SPTBN1 appear to connect a number of genes and these genes in turn confer to those biological functions and pathways which appear to control immune systems (Figure 3A). In addition, we have also analyzed the overall DEGs network (from all the datasets). Here, the top ranked genes are FN1, FAP, KANK4, COMP, FAM101B, COL1A2, ANKRD1, TAGLN, SPARC, ADAM19, OLFM4, CXCL10/11, OASL, FOS, APOBEC3A, IFI44L, IFI27, IFIT1, and RSAD2.

Prediction of putative drug-targets in case of COVID-19 infection: Finally, we performed docking studies for the top-ranked genes based on networks of DEGs by using three herbal drugs apigenin, quercetin, and resveratrol by using swiss-dock webserver (Figure 3). Here, the drugs were three: apigenin, quercetin, and resveratrol and five proteins: CBX3, SRSF6, DDX17, NDUFS1, and HECTD1. In the docking results, we observe that the three drugs bind almost in the same binding region where quercetin has the better Gibbs free energy (delta G or ΔG) i.e., −7.29 kcal/mol while the other two drugs have very close ΔG in case of CBX3. In DDX17, apigenin, quercetin, and resveratrol show ΔG −7.15, −7.31, and −7.51 kcal/mol, respectively, with NDUFS1 −7.03, −7.35, and −7.05 kcal/mol, respectively, with HECTD1, −7.26, −7.61, and −7.54 kcal/mol, respectively, and with SRSF6, −7.05, −6.63, and −6.73 kcal/mol, respectively. Here, it could be concluded that these three herbal drugs set display better binding possibilities in terms of ΔG.

## Discussion

Coronaviruses are a virus family that infects a variety of animals, including camels and bats. In rare situations, they can evolve and infect humans, transmitting the sickness from person to person. MERS—CoV and SARS—CoV are two more recent human coronaviruses associated to significant sickness. 1) 229E (alpha), 2) NL63 (alpha), 3) OC43 (beta), 4) HKU1 (beta), and 5) SARS—CoV are the other five coronaviruses (Djalante et al., 2020; Hamid et al., 2020; Laxminarayan et al., 2020). Respiratory and intestinal infections are the most common diseases in terms of clinical significance, but hepatic and neurological diseases can also occur. An outbreak like the one in Wuhan, China, could be caused by a variety of circumstances. The first and most crucial aspect is the anticipated growth of Wuhan’s seafood market, which was forecasted based on preliminary research. As a result, we can infer that the virus is transmitted from animals to people (Murrow and Debnath 2013; Cao et al., 2020; Jackson et al., 2020; Sharin et al., 2020).

Services for diagnosis and treatment should be offered across the country, especially in high-risk areas. Massive public awareness campaigns about the 2019-nCoV infection should be carried out on a large scale in order to inform the general public about the preventative measures that should be taken. Another factor connected to human transmission is frequent animal-human interactions (Boulware et al., 2020; Hassan et al., 2020; Hu et al., 2020; Moujaess et al., 2020; Wiersinga et al., 2020; Zhou et al., 2020).

The fact that a variety of factors influence a wide spectrum of clinical symptoms, from mild to severe, complicates its pathogenesis. Coronavirus can infect a range of organs and cell types, including gut mucosal cells, kidney epithelial cells, neurological systems, and the lymphatic system in general. Only one complete 2019-nCoV genome is currently available in GenBank, with the accession number MN908947 and a length of 29870-bp excluding the poly-A-tail. The five typical ORFs discovered on the same coding strand thus far are ORF1ab polyprotein (7096-aa), spike glycoprotein (1273-aa), envelope protein (75-aa), membrane protein (222-aa), and nucleo-capsid protein (419-aa). Bat SARS-like coronavirus appears to be the closest relative of 2019-nCoV, a new member of the Betacoronavirus genus with which it shares 88 percent nucleotide similarity (Laxminarayan et al., 2020; Wiersinga et al., 2020; Xu et al., 2020; Zhang et al., 2020). At the moment, transmission is a major challenge and a major emphasis in disease biology and infectious illness epidemiology. The virus’s control and coordination mechanisms have yet to be uncovered, however new research suggests that pathogenesis and amyloid production are driven by the viral protein corona (a protein covering adhering to its surface).

This research primarily focuses on gene expression profiling in order to better understand the impact of coronavirus in human cell lines. Unlike previous research, which focused on the impact of infection on the inflammatory system, we investigated the overall DEGs and their impact on a variety of pathways and functions, as well as presenting the selected genes with their networks based on their connectivities, fold changes, *p*-values, and the pathways they control. FN1, NFKBIA, FAP, KANK4, COMP, FAM101B, COL1A2, ANKRD1, TAGLN, SPARC, ADAM19, OLFM4, CXCL10/11, OASL, FOS, APOBEC3A, IFI44L, IFI27, IFIT1, and RSAD2 are among the top-ranked genes. This research will serve as a watershed moment in the search for or development of potential medicinal medications.

The pathways that were most typically enriched were largely part of the biological system that controls infection and inflammation, as well as a major portion of the immune system. As a result of COVID-19 replication in the cytoplasm of the host cell, the immune system, infection, and inflammatory systems are the key systems affected as a result of coronavirus invasion. The leading genes which are affected as a result of coronavirus replication within the cell line cytoplasm are NFKBIA, FN1, KANK4, FAP, TAGLN, ADAM19, ANKRD1, TAGLN, SPARC, ADAM19, OLFM4, CXCL10/11, OASL, FOS, APOBEC3A, IFI44L, IFI27, IFIT1, and RSAD2 and the functional changes are mainly associated with these pathways TNF, cytokine, NF—kB, TLR, TCR, BCR, FOXO, and TGF signaling pathways are among them and there are additional pathways such as hippo signaling, apoptosis, estrogen signaling, regulating pluropotency of stem cells, ErbB, Wnt, p53, cAMP, MAPK, PI3K—AKT, oxidative phosphorylation, protein processing in endoplasmic reticulum, prolactin signaling, adipocytokine, neurotrophine signaling, and longevity regulating pathways. In terms of exploring the possible herbal drugs against COVID-19 infection, we have explored apigenin, quercetin, and resveratrol against the genes which appear significant i.e., NDUFS1, SRSF6, HECTD1, CBX3, and DDX17. We feel that this study provides straight-forward in-silico analysis of COVID-19 infection followed by the possible biomarkers and the herbals drugs and could be extended to *in-vitro* and *in-vivo* study in terms of future perspective and the same is the limitations of this study.

## Conclusion

Based on our studies, we conclude that the most typically impacted genes as a result of coronavirus replication within the cell line cytoplasm are NFKBIA, FN1, KANK4, FOXO3, ANKRD1, TAGLN, SPARC, ADAM19, OLFM4, CXCL10/11, OASL, FOS, APOBEC3A, IFI44L, IFI27, IFIT1, and RSAD2. The pathways which appear to be primarily responsible for the functional alterations were TNF, cytokine, NF—kB, TLR, TCR, BCR, Foxo, and TGF signaling pathways and the additional pathways were also dominant and those pathways are hippo signaling, apoptosis, oestrogen signaling, regulating pluripotency of stem cells, ErbB, Wnt, p53, cAMP, MAPK, PI3K—AKT, oxidative phosphorylation, and protein processing in the endoplasmic The three natural medications apigenin, quercetin, and resveratrol may be viable therapeutic candidates against COVID-19 infection in terms of docking.

## Data Availability

The datasets presented in this study can be found in online repositories. The names of the repository/repositories and accession number(s) can be found in the article/Supplementary Material.
