# Supplementary material for: Global Gene Expression and Docking Profiling of COVID-19 Infection
Source: Front Genet. 2022 Apr 11;13:870836. doi: 10.3389/fgene.2022.870836 (PMC9035897; doi:10.3389/fgene.2022.870836)
Supplement: Supplementary file 1 [file Table1.DOCX]

Table 1. Enriched pathways for GSE183071 COVID-19 dataset DEGs.

| **Enriched Pathways** | **p-values** |
| --- | --- |
| KEGG_04010_MAPK_signaling_pathway | 5.42E-20 |
| KEGG_04060_Cytokine-cytokine_receptor_interaction | 5.42E-20 |
| KEGG_04064_NF-kappa_B_signaling_pathway_-_Homo_sapiens_(human) | 5.42E-20 |
| KEGG_04145_Phagosome | 5.42E-20 |
| KEGG_04151_PI3K-Akt_signaling_pathway_-_Homo_sapiens_(human) | 5.42E-20 |
| KEGG_04210_Apoptosis | 5.42E-20 |
| KEGG_04380_Osteoclast_differentiation | 5.42E-20 |
| KEGG_04514_Cell_adhesion_molecules_(CAMs) | 5.42E-20 |
| KEGG_04612_Antigen_processing_and_presentation | 5.42E-20 |
| KEGG_04620_Toll-like_receptor_signaling_pathway | 5.42E-20 |
| KEGG_04630_Jak-STAT_signaling_pathway | 5.42E-20 |
| KEGG_04640_Hematopoietic_cell_lineage | 5.42E-20 |
| KEGG_04650_Natural_killer_cell_mediated_cytotoxicity | 5.42E-20 |
| KEGG_04660_T_cell_receptor_signaling_pathway | 5.42E-20 |
| KEGG_04662_B_cell_receptor_signaling_pathway | 5.42E-20 |
| KEGG_04668_TNF_signaling_pathway_-_Homo_sapiens_(human) | 5.42E-20 |
| KEGG_04068_FoxO_signaling_pathway_-_Homo_sapiens_(human) | 4.11E-19 |
| KEGG_04722_Neurotrophin_signaling_pathway | 4.11E-19 |
| KEGG_04917_Prolactin_signaling_pathway | 1.56E-16 |
| KEGG_04920_Adipocytokine_signaling_pathway | 1.56E-16 |
| KEGG_04015_Rap1_signaling_pathway_-_Homo_sapiens_(human) | 4.78E-14 |
| KEGG_04810_Regulation_of_actin_cytoskeleton | 4.78E-14 |
| KEGG_04014_Ras_signaling_pathway_-_Homo_sapiens_(human) | 7.65E-13 |
| KEGG_04066_HIF-1_signaling_pathway_-_Homo_sapiens_(human) | 7.65E-13 |
| KEGG_04670_Leukocyte_transendothelial_migration | 7.65E-13 |
| KEGG_04071_Sphingolipid_signaling_pathway_-_Homo_sapiens_(human) | 1.15E-11 |
| KEGG_04510_Focal_adhesion | 1.15E-11 |
| KEGG_04621_NOD-like_receptor_signaling_pathway | 1.15E-11 |
| KEGG_04550_Signaling_pathways_regulating_pluripotency_of_stem_cells | 1.61E-10 |
| KEGG_04610_Complement_and_coagulation_cascades | 1.61E-10 |
| KEGG_04622_RIG-I-like_receptor_signaling_pathway | 1.61E-10 |
| KEGG_04370_VEGF_signaling_pathway | 2.09E-09 |
| KEGG_04611_Platelet_activation | 2.09E-09 |
| KEGG_04672_Intestinal_immune_network_for_IgA_production | 1.58E-08 |
| KEGG_04664_Fc_epsilon_RI_signaling_pathway | 2.51E-08 |
| KEGG_04115_p53_signaling_pathway | 2.76E-07 |
| KEGG_04360_Axon_guidance | 2.76E-07 |
| KEGG_04919_Thyroid_hormone_signaling_pathway | 2.76E-07 |
| KEGG_04921_Oxytocin_signaling_pathway | 2.76E-07 |
| KEGG_04012_ErbB_signaling_pathway | 2.76E-06 |
| KEGG_04072_Phospholipase_D_signaling_pathway_-_Homo_sapiens_(human) | 2.48E-05 |
| KEGG_04310_Wnt_signaling_pathway | 2.48E-05 |
| KEGG_04350_TGF-beta_signaling_pathway | 2.48E-05 |
| KEGG_04392_Hippo_Signaling_Pathway | 2.48E-05 |
| KEGG_04520_Adherens_junction | 2.48E-05 |
| KEGG_04750_Inflammatory_mediator_regulation_of_TRP_channels | 2.48E-05 |
| KEGG_04623_Cytosolic_DNA-sensing_pathway | 1.08E-04 |
| KEGG_04110_Cell_cycle | 1.98E-04 |
| KEGG_04512_ECM-receptor_interaction | 1.98E-04 |
| KEGG_04912_GnRH_signaling_pathway | 1.98E-04 |
| KEGG_03050_Proteasome | 1.39E-03 |
| KEGG_04024_cAMP_signaling_pathway_-_Homo_sapiens_(human) | 1.39E-03 |
| KEGG_04211_Longevity_regulating_pathway | 1.39E-03 |
| KEGG_04540_Gap_junction | 1.39E-03 |
| KEGG_04666_Fc_gamma_R-mediated_phagocytosis | 1.39E-03 |
| KEGG_04910_Insulin_signaling_pathway | 1.39E-03 |
| KEGG_04915_Estrogen_signaling_pathway | 1.39E-03 |
| KEGG_00230_Purine_metabolism | 8.33E-03 |
| KEGG_04022_cGMP-PKG_signaling_pathway_-_Homo_sapiens_(human) | 8.33E-03 |
| KEGG_04120_Ubiquitin_mediated_proteolysis | 8.33E-03 |
| KEGG_04140_Regulation_of_autophagy | 8.33E-03 |
| KEGG_04141_Protein_processing_in_endoplasmic_reticulum | 8.33E-03 |
| KEGG_04371_Apelin_signaling_pathway_-_Homo_sapiens_(human) | 8.33E-03 |
| KEGG_04916_Melanogenesis | 8.33E-03 |
| KEGG_04020_Calcium_signaling_pathway | 4.17E-02 |
| KEGG_04080_Neuroactive_ligand-receptor_interaction | 4.17E-02 |
| KEGG_04144_Endocytosis | 4.17E-02 |
| KEGG_04261_Adrenergic_signaling_in_cardiomyocytes | 4.17E-02 |
| KEGG_04530_Tight_junction | 4.17E-02 |
| KEGG_04723_Retrograde_endocannabinoid_signaling | 4.17E-02 |
| KEGG_04914_Progesterone-mediated_oocyte_maturation | 4.17E-02 |
